# Mixed methods research on satisfaction with athletes’ compensation

**DOI:** 10.1038/s41598-024-55297-x

**Published:** 2024-02-24

**Authors:** Qianqian Li, Shuo Li, Huan Zhao, Lu Jiao, Xiao Han

**Affiliations:** 1 School of Management, Beijing Sport University, Beijing, 100084 China; 2 Science and Technology Branch, Shandong Sport University, Jinan, 250102 China; 3 School of Sports Science, Honam University, Gwangju, 62399 Korea; 4 Foundation Department, Shandong Xiehe University, Jinan, 250013 Shandong China; 5 Faculty of Physical Education, Tomsk State University, Tomsk, 634050 Russia

**Keywords:** Athlete, Compensation satisfaction, Mixed research method, Regression analysis, QCA, Human behaviour, Psychology

## Abstract

This study explores the factors influencing athletes’ compensation satisfaction and their configuration effects. A mixed research approach that combines regression analysis and fuzzy-set qualitative comparative analysis (fsQCA) was applied to process the survey data of 352 athletes from six provinces. The regression analysis results demonstrate that economic compensation, the compensation system, the external environment, and compensation fairness all have a significant positive effect on athletes’ compensation satisfaction. Accordingly, the fsQCA approach was applied to explore the combined effects of the factors influencing athletes’ compensation satisfaction, which verifies the conclusions drawn from regression analysis and provides improvement paths for increasing athletes’ compensation satisfaction.

## Introduction

Compensation satisfaction is an indicator of employee recognition, dedication, and turnover rate^1^. It is crucial to individual development in the organization as well as the growth of the team. As Chinese-style sports undergo modernization, competitive sports face a paradigm shift, necessitating adherence to evolving standards. Consequently, the study of athletes' compensation satisfaction, a pivotal driving force behind competitive sports' success, is gaining increasing traction within academic discourse. However, athletes’ remuneration in China varies, and significant differences exist in the remuneration methods and pay ratios of athletes in different sports. Athletes generally have negative attitudes toward the overall remuneration and bonus distribution^2^, which severely affects their training status and motivation and deviates from the steady move toward modernizing Chinese-style competitive sports. In this context, exploring the influencing factors of athletes’ compensation satisfaction is of great significance for improving the structure and system of athletes’ compensation distribution, as well as standardizing the system of athletes’ compensation distribution to increase the fairness of athletes’ income distribution and steadily improve Chinese competitive sports. Therefore, this study conducts a mixed study to explore in depth the influencing factors that affect athletes’ compensation satisfaction, analyzes the influence of the combination conditions among the influencing factors on athletes’ compensation satisfaction, and proposes feasible suggestions for athletes’ compensation satisfaction.

## Literature review

### Review of the factors influencing athletes’ compensation satisfaction

Compensation or wage, in a narrow sense, refers to money paid to workers. With the continuous development of wage, human capital, and incentive theories, its definition has extended beyond material compensation such as wages, bonuses, benefits, and dividends^3^, to include spiritual benefits, such as spiritual satisfaction, development space, training, and further education^4^. Compensation can motivate employee behavior, so organizations must ensure employees are satisfied with compensation^5^. Therefore, one of the important issues in organizational management is how to improve employee compensation satisfaction and enhance employee motivation to work, thereby creating greater organizational performance. The theory of difference suggests that, compensation satisfaction refers to the employee’s perception of the difference between the expected and actual incomes^6^. It is the sentiment associated with comparing the actual situation with the desired goal^7^. In addition, equity theory suggests that the actual level of an individual's income is not the key to pay satisfaction, it is the comparison of input–output ratios between individuals and others that is central to determining pay satisfaction^8^.

The current research on compensation satisfaction focuses on dimensions and measurement. (1) Regarding the dimensions of compensation satisfaction, many scholars believe that the compensation level, benefits, compensation increase, and compensation structure^9,10^. In addition, recognition, flexible job design, organizational justice, job security, and organizational motivation^11,12^, compensation management, monetary compensation, pay equity, compensation system (CS), work environment, and job value affect compensation satisfaction^13,14^. The meta-analysis results of Williams^15^ also showed that salary level is an important but not decisive factor affecting compensation satisfaction. A well-designed salary system can improve satisfaction, recruit and retain outstanding talents, and thus generate a competitive advantage^16^. It can also promote internal and external salary fairness, employees with lower salary levels are more satisfied with their salary levels than those with higher levels^17^. Even if economic compensation is high, if employees perceive unfair salary distribution, their compensation satisfaction may also decrease. On the contrary, if employees believe that compensation is fair, even if economic compensation is not the highest, their satisfaction may still be higher^18^. In addition, another part of compensation satisfaction may be due to the employer's ability to meet employees' non-financial needs and expectations. Positive behaviors such as employee engagement and commitment are the result of organizational environmental conditions, including policies requiring management to recognize outstanding employees^19^. A good work environment can affect employees' multiple positive behaviors, even if the salary is relatively low, appreciating and recognizing employees' efforts can also increase their engagement, thereby improving their compensation satisfaction. In summary, these four factors are interdependent and mutually influencing, forming an important component of the complex network that affects employee compensation satisfaction. A well-designed compensation system can ensure the reasonable distribution of economic compensation, promote compensation fairness, and promote the organization to adapt to changes in the external environment, thereby improving employee compensation satisfaction. (2) Regarding compensation satisfaction measurement, the current research mostly adopts quantitative analysis methods^20^, such as multiple regression models and the structural equation model^21,22^. Focusing on the net effect of a single influencing factor makes detecting the joint effect of multiple factors difficult. In addition, some scholars have adopted theoretical and descriptive analyses to evaluate the impact of CS changes on compensation satisfaction^22^, but more practical data are needed for support.

Researchers have discussed the current state of athlete compensation satisfaction and explored the factors influencing it from the perspective of weighted factors^23,24^. Some scholars suggest that the implementation of appropriate compensation system can stimulate the motivation of athletes, and the survey shows that unfair treatment, cognitive dissonance and the willingness to leave are important factors affecting the positive performance of athletes^25^. Taking professional football players in major Italian leagues as an example, the relationship between personal productivity and salary can lead to the superstar effect, where the best players, including superstars, are attracted to the highest paying teams and leagues^26^. The contribution of individual player performance to team performance is a direct factor in salary differences. In addition to individual skills, team attributes (the interaction between team values and player attributes) are also important factors affecting player compensation^27^. From the perspective of the compensation incentive effect of athletes, direct economic compensation satisfaction, direct non-economic compensation satisfaction, and indirect non-economic compensation satisfaction have a significant positive impact on the compensation incentive effect of athletes. Direct economic compensation satisfaction has the greatest contribution to the influencing factors of compensation incentive effect^28^. Chinese athletes are a special group, a national role model, and an important component of society. They also enjoy the same development rights as the public, and cannot ignore the reality and needs of athletes as "social people", nor can they separate the interaction between athletes and the external environment of society. These characteristics determine their diverse needs, such as the desire for good salaries and benefits, the need to meet national honor, realize self-worth, obtain good development opportunities, and harmonious interpersonal relationships. However, in reality, Chinese professional athletes face low salary performance and a relatively lagging salary system, which includes the rapid development of the leisure and entertainment industry due to macroeconomic growth, as well as institutional factors left over from the planned economy, as well as cultural and social factors^29^. Therefore, economic compensation, compensation system, and external social environment are important factors that affect the satisfaction of Chinese athletes' compensation. In addition, only fairness can enable athletes to adhere to the most basic industry norms, efficiency can improve the value of human and social resources, and promote industry development^30^.

### Research gaps and directions

Previous studies have explored various aspects of compensation satisfaction, which provide a theoretical basis for this study. However, the following gaps still exist: (1) most scholars have conducted comprehensive studies on the influencing factors of compensation satisfaction in various fields. The respondents of the study were mostly employees of companies^19,31^, technologists^14,32^, teachers^21,33^ and hospitals^34,35^. However, they have not focused enough on athletes’ compensation satisfaction. Only a few expert studies, Especially Chinese athletes^2,24,28,36^. More research that focuses specifically on athletes' compensation satisfaction is needed. The object of study in this manuscript is the salary satisfaction of Chinese athletes, which can make up for the lack of current research to a certain extent. (2) However, research on the influencing factors of athletes’ compensation satisfaction is not comprehensive and their research focuses on the history of athlete compensation^22,37^ and analyses of issues^23,38^. Only a few scholars have explored athletes' pay satisfaction, using structural equation modelling, factor analysis and non-parametric tests to analyzed the weighting of factors influencing athletes' pay (either individually or in specific combinations)^24,27,28^. Existing research on athlete compensation has not adequately considered the "joint effect" of multiple factors. For instance, the influencing factors of athletes’ compensation satisfaction and whether a complex relationship exists between the influencing factors are still an unknown black box. Notably, mixed methods((fsQCA) can be used to support the "joint effects" of multiple factors and the "interactions" between multiple factors behind the case, and it is considered to be an effective way to explore the joint effects and interactions behind things^39^. It can solve the causal complexity problem that cannot be solved by the variable-oriented large sample analysis through the overall group analysis, deconstruct the mechanism that generates the complex sports social phenomenon, and propose the combination path. In the field of sports, some scholars have explored the possible paths for the realisation of mass participation satisfaction, arguing that QCA can better understand and predict the decision-making process of sports consumers, providing a new way to improve athletes' satisfaction^40,41^. Athletes, as a special group, have multiple subjective individual logics, and the development of their remuneration is affected by multiple factors such as politics, economy, culture, society, individuals, etc., and the relationship between the influences is complicated, which is in line with the research object of fsQCA "multi-causal induced". The fsQCA has its own unique advantages in solving this kind of problems, which can accurately identify the core influencing factors and make clear the inner correlation between them, which is of great significance to the national pay system as a whole. Therefore, this paper applies a mixed research approach (fsQCA), based on the personal characteristics of athletes, questionnaire survey results, and Chen Tao's^14^ compensation satisfaction dimension structure, combined with the qualitative research results of the influencing factors of athlete salary, to examine and analyze four factors affecting compensation satisfaction: economic compensation (EC), compensation system (CS), external environment (EE), and compensation fairness (CF) based on the characteristics of athletes. It provides an important methodological underpinning for the study of athlete compensation issues. (3) Previous studies were mostly based on scholars' subjective analyses, lack of quantitative data collection and analysis of research and small sample sizes^23,25^, research on athlete compensation satisfaction needs to be more comprehensive. Specifically, this manuscript collects a sample size of 352 surveys, examines the interplay of various factors of athletes' pay satisfaction through quantitative methods, and conducts qualitative comparative analyses fsQCA of them with a view to proposing scientifically sound development recommendations. It breaks through the one-sidedness of previous subjective studies and increases the sample size of athletes, thus enriching the database of the athlete population and providing a more comprehensive support for future research on athletes.

## Research hypothesis

### Economic compensation

EC refers to the basic salary, bonus income, and welfare treatment. It includes direct compensation in the form of salaries, wages, bonuses, and commissions received by individuals as well as indirect EC. Theoretically, the higher the EC, the higher the satisfaction will be. Improving EC is seen in some areas as a key factor to improving quality and attracting and retaining quality talent^42,43^. Moreover, increasing the basic salaries and improving the EC package of employees can significantly improve their satisfaction^34^. Especially in less developed countries, low EC levels are considered the most important factors affecting compensation satisfaction^33^. It is clear that compensation is not only a reward for athletes, but also plays an important role in conveying information to athletes and guiding behavior, especially economic compensation, which can drive positive performance. There are various differences and unfairness in the economic compensation of Chinese athletes, and there are a lot of factors that cause the differences in economic compensation, such as differences in gender, length of service, age, rank and other individual characteristics, Only by understanding the role of economic compensation in compensation satisfaction and its relationship with other variables that influence it, can we provide concrete solutions to optimize the criteria for setting economic compensation and improve athletes’ compensation satisfaction. Therefore, our assumption is that.

H1: the higher the economic compensation, the higher the athletes’ satisfaction.

### Compensation system

CS is the organization’s system for distributing compensation, which includes income distribution, promotion, and reward and punishment systems. CS reform can improve employees’ compensation satisfaction^35^ and provide a foundation for exchange relationships between individuals and organizations. Moreover, its significance extends beyond job compensation^12^. The reward and punishment system is an important part of CS reform, whose basic purpose is to attract and retain employees and influence them to make decisions that support organizational goals, improve organizational efficiency through quality and performance improvement, and align services and organizational added value^44,45^. Therefore, our assumption is that.

H2: the compensation system has a positive effect on athletes’ compensation satisfaction.

### External environment

EE is mainly reflected in how much the organization values its employees and their work, which includes social status, leaders’ attention, and training conditions. The EE, nature of work, and communication are reported to influence job pay satisfaction. “Companies need to be aware of the importance of a good external environment to maximize job compensation satisfaction”^46–48^. The absence of these key aspects may lead to lower job compensation satisfaction, negatively affecting the productivity of employees and their organizations^49^. In addition, employee recognition, which is external to the work environment, can be considered a precursor of compensation satisfaction^19^. Therefore, our assumption is that.

H3: the better the external environment, the higher the athletes’ compensation satisfaction.

### Compensation fairness

CF refers to the ratio between what employees deliver to the organization and the compensation they receive^50^, including internal equity and external equity. Social comparison of compensation affects compensation satisfaction, and compensation comparison affects compensation satisfaction by influencing the sense of fairness, with distributional fairness (internal vs. external) being more influential than procedural fairness^17^. Therefore, CF has a significant influence on compensation satisfaction and can improve the work engagement and satisfaction of employees^51,52^. Therefore, our assumption is that.

H4: the higher the compensation fairness, the higher the athletes’ compensation satisfaction.

## Research process and methods

### Questionnaire design

The questionnaire consisted of (a) relevant demographic information, including gender, age, training years, sport group, sport level, and region of affiliation and (b) athletes’ perceptions and satisfaction with their salaries. Considering relevant sources^24^, the second part contained 13 items of satisfaction measurement. A 5-point Likert scale was used to measure athletes’ salary satisfaction; “1” (strongly dissatisfied), “2” (somewhat dissatisfied), “3” (neither satisfied nor dissatisfied), “4” (somewhat satisfied), and “5” (strongly satisfied). Details on these items can be found in Table 1.

**Table 1:** Athletes' compensation satisfaction survey questionnaire.

Latent variables	Measurement items	Question items
Compensation satisfaction (Y)	Satisfaction	How satisfied are you with your compensation overall?
Economic compensation (EC)	Basic salary (X1)	Are you satisfied with your current basic salary?
	Bonus income (X2)	Are you satisfied with the current bonus income?
	Welfare treatment (X3)	Are you satisfied with the current welfare?
Compensation system (CS)	Income distribution system (X4)	Do you think the current income distribution system incentive effect?
	Promotion system (X5)	Do you think the current promotion system is perfect?
	Reward and punishment system (X6)	Do you think the current reward and punishment system is perfect?
External environment (EE)	Social status (X7)	Are you satisfied with the current recognition of athletes’ social status?
	Leaders' attention (X8)	Are you satisfied with the importance your leaders place on you or the team?
	Training conditions (X9)	Are you satisfied with the physical, social and emotional aspects of the training environment?
Compensation fairness (CF)	Personal fairness (X10)	Do you think you give as much as you get in return?
	Internal fairness (X11)	Do you think the current income gap between team members is reasonable?
	External fairness (X12)	Do you think the income gap between different sports is reasonable?

Cronbach’s Alpha coefficient was calculated for the data on each variable based on the observed variables of athletes’ compensation satisfaction and determined to be 0.715. This indicated that the questionnaire scale design had high reliability and consistency. Exploratory factor analysis (EFA) was analyzed using KMO (Kaiser–Meyer–Olkin) and Bartlett's test of spheres to determine the suitability of the data collected for factor analysis. The significant value < 0.001 indicates that a factor analysis may be worthwhile for the data set. In addition, the KMO and Bartlett's Spherical test results were 0.745 and 1940.113, respectively, indicating that the questionnaire was suitable for factor analysis. With principal component analysis, the factors were further selected by applying Varimax with eigenvalues greater than 1. A total of five common factors were extracted, explaining a total of 78.67% of the variance. This indicates that the validity of the questionnaire is good.

### Data collection and processing

#### Data collection and description

A self-report questionnaire was used to collect data for this study. According to the Declaration of Helsinki, all subjects gave written informed consent. Survey subjects were assured confidentiality and anonymity. All participation was voluntary. Ethical approval was provided by the Beijing Sport University Ethics Committee of Sport (Project number: 2021124H). This study was conducted in accordance with relevant guidelines and regulations.

The sample size was 5–10 times the number of items, which should be about 130, since there were 13 items in this study. G*power calculations resulted in a sample size of 134 which was enough for this study. Therefore, a sampling method was used to randomly select six provinces, Guangdong, Liaoning, Hunan, Heilongjiang, Qinghai and Yunnan, representing the eastern, central and western regions, and 400 questionnaires were distributed and 390 were returned by taking the opportunity of intensive training for sports teams. There were 352 valid questionnaires, with a validity rate of over 90%.The questionnaire data revealed that the number of male athletes was 1.5 times higher than that of females. The number of those under the age of 21 was approximately the same as those over 21. Athletes with less than 5 years of training experience (63.6%) were the majority. Moreover, national level athletes and specialized athletes were the majority, 45.7% and 64.8%, respectively. Regionally, the selected athletes were from the eastern, central, and western regions of China, with the majority being athletes from the eastern region (72.4%). Notably, the distribution of sample objects was reasonable, as listed in Table 2.

**Table 2:** Characteristics of the sample (N = 352).

Information	Category	Frequency	%
Gender	Male	213	60.5
	Female	139	39.5
Age	< 21	166	47.2
	≥ 21	186	52.8
Training years	< 5	224	63.6
	≥ 5	128	36.4
Level	Master sportsman	158	44.9
	National-level athletes	161	45.7
	Second-level athletes	33	9.4
Group	specialized athletes	228	64.8
	Professional athlete	66	18.8
	semiprofessional athletes	58	16.5
Region	Eastern region	255	72.4
	Central region	38	10.8
	Western region	59	16.8

### Research methods

The mixed method utilized in this study is a combination of regression analysis (quantitative) and QCA (qualitative). First, regression analysis was used to examine the direction, degree, and ranking of each influencing factor on athletes’ compensation satisfaction^53^. Second, qualitative analysis (QCA) was applied to examine the conditional factors and their combined effects on athletes’ compensation satisfaction^54,55^. SPSS26.0 software and fsQCA3.0 software were used to perform regression analysis and QCA, respectively.

## Analyses and results

### Regression analysis

The multiple linear regression model was used to determine the relationship between the dependent variable Y and the independent variable X_i_ and to establish a linear regression equation with the parameters of the independent variables and the disturbance terms to predict the dependent variable^56^. The regression model is set up as follows:

$$Y = \beta_{0} + \beta_{1} \chi_{1} + \beta_{2} \chi_{2} + ...\beta_{i} \chi_{i} + \varepsilon$$

In the above equation, Y denotes the dependent variable, *β*_i_ is the undetermined parameter, X_i_ is the independent variable, and ε is the disturbance factor. A stepwise regression approach was applied to analyze the relationship between athletes’ compensation satisfaction and each influencing factor, namely EC, CS, EE, and CF. The factors with significant effects were selected as independent variables, and the optimal regression equation was established and then analyzed to determine the relationship between the independent and dependent variables.

As presented in Table 3, the complex correlation coefficient R in the final regression model was 0.657, indicating a good model fit. The determination coefficient R^2^ was 0.432, indicating that the independent variables can explain 43.2% of the variation in the dependent variable. The DW statistic was 1.872, which is close to 2^57^, indicating that the autocorrelation among the independent variables is not pronounced and independent. Therefore, they can be subjected to stepwise regression analysis.

**Table 3:** Summary of determination coefficients of stepwise regression models.

Model	R	R^2^	Adjusted R^2^	SEE	DW
1	0.310^a^	0.096	0.094	0.988	
2	0.402^b^	0.162	0.157	0.9535	
3	0.533^c^	0.284	0.277	0.882	
4	0.657^d^	0.432	0.425	0.787	1.872

*SEE* standard error of estimate, *DW* Durbin–Watson.

As listed in Table 4, the tolerance values of the covariance statistics in the final regression model are all close to 1 and the variance inflation factor (VIF) are all close to 1, indicating that none of the variables in the model have multicollinearity problems with each other^57^.

**Table 4:** Comparison of stepwise regression coefficients.

Model		Unstandardized coefficients		Standardized coefficients	t	Sig	Collinearity statistics	
		B	SE	Beta			T	VIF
1	Constant	1.240	0.165		0.000	1.000		
	Economic compensation	0.583	0.064	0.436	9.069	0.000	1.000	1.000
2	Constant	0.751	0.192		0.000	1.000		
	Economic compensation	0.514	0.064	0.385	8.010	0.000	0.947	1.056
	Compensation system	0.233	0.051	0.221	4.603	0.000	0.947	1.056
3	Constant	0.255	0.214		0.000	1.000		
	ECONOMIC COMPENSATION	0.482	0.063	0.361	7.694	0.000	0.936	1.069
	Compensation system	0.166	0.051	0.158	3.252	0.001	0.875	1.142
	External environment	0.281	0.059	0.229	4.780	0.000	0.898	1.113
4	Constant	− 1.121	0.239		0.000	1.000		
	Economic compensation	0.459	0.056	0.344	8.207	0.000	0.934	1.071
	Compensation system	0.135	0.046	0.128	2.960	0.003	0.871	1.148
	External environment	0.216	0.053	0.176	4.088	0.000	0.883	1.132
	Compensation fairness	0.633	0.066	0.392	9.514	0.000	0.965	1.037

*SE* Std. error, *T* tolerance;

As presented in Tables 3 and 4, the stepwise regression yielded Model 1, Model 2, Model 3, and Model 4 with a gradual increase in R^2^ and adjusted R^2^ of 0.432 and 0.425, respectively. The regression results clarify that EC, CS, EE, and CF all have a significant positive effect on compensation satisfaction. Among them, pay fairness had the largest effect on pay satisfaction (β = 0.392, *p* < 0.001), indicating that improving pay fairness can improve athletes’ compensation satisfaction.

### Qualitative comparative analysis

To identify configurations in phenomena reliably, QCA is applied to estimate the causal contribution of various possible configurations to the expected outcome. Among the main variants of QCA are crisp-set QCA, multivalue QCA, and fuzzy set-based QCA^58^, we focus on fsQCA, one of the most general versions of QCA that does not significantly increase the computational cost of performing the analysis.

#### Data calibration

The Likert 5-point scale was utilized in this study. Although the degree of variables was differentiated during design, the scores may be influenced by some subjectivity because the questionnaire was self-rated. Therefore, this study applied the direct method to calibrate all variables. Using fsQCA 3.0 computing software, the scores of each variable were converted into fuzzy affiliation scores with values ranging from 0 to 1: three critical values were set for complete nonaffiliation, crossover point, and complete affiliation, with variable anchor points of 5%, 50%, and 95% of the sample, respectively^59^. The anchor points of each conditional variable are listed in Table 5.

**Table 5:** Calibration of fuzzy sets.

VARIABLES	0.95 (fully in)	0.5 (crossover)	0.05 (full out)
Economic compensation	3.67	2.33	1.33
Compensation system	4.67	2.67	1.33
External environment	4	3	1
Compensation fairness	3.67	2.67	1.67
Compensation satisfaction	4.5	2.5	1

#### Necessity analysis

Before the formal QCA analysis was conducted, an analysis of the necessity of each antecedent condition variable on the outcome variable was first conducted. The data from the study are listed in Table 6: the consistency of all antecedent conditions did not reach 0.9. Thus, none of the individual antecedent conditions (EC, CS, EE, CF) constituted a necessary condition for the outcome variable (compensation satisfaction), indicating that the individual condition variables were weak in explaining satisfaction with athlete compensation. Therefore, the conditional variables were in turn included from the truth table analysis to further explore the groupings that produced high satisfaction.

**Table 6:** Necessity test results.

Conditions tested	Consistency	Coverage	Conditions tested	Consistency	Coverage
Economic compensation (EC)	0.748	0.753	~ Economic compensation (EC)	0.556	0.596
Compensation system (CS)	0.715	0.736	~ Compensation system (CS)	0.592	0.620
External environment (EE)	0.673	0.769	~ External environment (EE)	0.646	0.614
Compensation fairness (CF)	0.758	0.766	~ Compensation fairness (CF)	0.554	0.592

∼, absence of condition;

#### Configuration analysis

This study followed the recommendations of Fiss^59^ and Xin^60^ and set the frequency threshold to 1, the consistency threshold to 0.8, and the proportional reduction in inconsistency (PRI) to 0.7 to perform a standard analysis on the samples. The corresponding complex, simple, and intermediate solutions were obtained. Because the intermediate solution only contains logical residuals that are consistent with theoretical logic and practical knowledge and does not allow for the elimination of necessary conditions, it is usually better than the simple and complex solutions. Therefore, this study combines the simple and intermediate solutions to further investigate the influence path of athlete compensation satisfaction. The conditions that appear in both the simple and intermediate solutions are considered core conditions (they have a significant impact on the outcome), whereas the conditions that only appear in the intermediate solution are considered auxiliary conditions (they have a secondary effect on the outcome).

The results in Table 7 reveal that, given our assumptions, two major configurations lead to intention to use. Their consistencies were 0.927 and 0.932, respectively, with a solution consistency of 0.918, which is greater than the threshold of 0.8. This indicates that the two configurations are sufficient conditions for compensation satisfaction, and equivalence exists. The solution coverage was 0.536, indicating that these two configurations can explain approximately 53.6% of the high satisfaction cases.

**Table7:** Configuration results.

	EC*CS*CF	EC*EE*CF
Economic compensation (EC)	●	●
Compensation system (CS)	●	
External environment (EE)		●
Compensation fairness (CF)	●	●
Raw coverage	0.473	0.464
Unique coverage	0.072	0.063
Consistency	0.927	0.932
Solution coverage	0.536	
Solution consistency	0.918	

Regarding the complete sample, Configuration 1 (EC*CS*CF) revealed that high satisfaction with athlete compensation is attributable to EC, CS, and CF. This solution has a consistency of 0.927 and explains a large number of cases (coverage = 0.473). Configuration 2 (EC*EE*CF) revealed that EC, EE, and CF promoted high satisfaction with athlete compensation. This solution explained 46.4% of the cases and had a consistency of 0.932. Thus, both solutions (Configuration 1 and 2) include EC and CF, which implies that these conditions are necessary to achieve high satisfaction with athlete compensation.

#### Robustness analysis

Three main methods are commonly applied to validate the robustness of the QCA method: (1) replacing the calibration anchor points, (2) adjusting the frequency of sample cases, and (3) increasing the consistency threshold^61^. In this study, the frequency of sample cases was increased to 2, the consistency threshold was increased to 0.85, and the PRI was increased to 0.75 for standard procedure analysis. The configuration results of athletes’ compensation satisfaction were consistent with those before adjustment.

## Conclusions and limitations

### Conclusions

The new and unique findings of this study are that examined the four factors (EC, CS, EE, and CF) influence athletes’ compensation satisfaction from a mixed-methods perspective using stepwise regression analysis and QCA. The results of the regression analysis confirmed that all the factors proposed in this study had a significant positive effect on athletes’ compensation satisfaction, with CF having the greatest effect, followed by EC, and then EE and CS. Therefore, managers should focus on CF and EC to improve athletes’ compensation satisfaction.

Based on the findings, our study suggested that: (1) although athletes have a strong desire for spirituality, organizational respect, and social recognition, basic material needs are essential. In a sense, the level of compensation income and its perceived fairness is an important reflection of the athletes’ own values and social status, so the CS should be improved to reflect the athletes’ labor value in the compensation. In addition to basic salary, performance and so on, consideration can also be given to providing athletes with benefits in the event of unemployment, retirement and so on. At the same time, it is also necessary to formulate plans for athletes to be promoted to coaches, career transition planning, etc., so as to help athletes have better career development after retirement. (2) CS is a top-level design of athlete compensation optimization, and an ideal system is crucial. As Zou^24^ said, "Focus on further improving the pay system and pay structure for different types of athletes at the level of the pay system, so as to enhance the satisfaction of athletes' compensation". However, in China training practice, athletes, especially the young ones, pay more attention to the level of compensation income, EE, and their perceived CF. Therefore, giving athletes strong financial support and an EE with competitive advantages can improve their pay satisfaction even if the payment system environment is not ideal.

### Limitations

This study has two limitations. On the one hand, the data sample and sources are limited. This study considers the Chinese national situation to analyze the factors influencing athletes’ compensation satisfaction. Therefore, the recommendations are somewhat situational. Although the sample includes athletes from different provinces and regions in China to increase the generalizability of the findings, the sample size can be expanded, and the sample can be scoped to include athletes from different countries. On the other hand, athletes’ compensation satisfaction is influenced by multidimensional factors. Individual differences among athletes were not considered in the analysis. Therefore, future research should focus on the effect of the different characteristics of athletes on compensation satisfaction.

## Data Availability

The datasets used and analyzed during the current study available from the corresponding author on reasonable request.
